# CD4+Foxp3+T Regulatory Cells Promote Transplantation Tolerance by Modulating Effector CD4+ T Cells in a Neuropilin-1-Dependent Manner

**DOI:** 10.3389/fimmu.2019.00882

**Published:** 2019-04-24

**Authors:** Mauricio Campos-Mora, Pamina Contreras-Kallens, Felipe Gálvez-Jirón, Masyelly Rojas, Carolina Rojas, Aarón Refisch, Oscar Cerda, Karina Pino-Lagos

**Affiliations:** ^1^Centro de Investigación Biomédica, Facultad de Medicina, Universidad de los Andes, Las Condes, Chile; ^2^Programa de Biología Celular y Molecular, Facultad de Medicina, Instituto de Ciencias Biomédicas (ICBM), Universidad de Chile, Santiago, Chile; ^3^Facultad de Odontología, Universidad de Chile, Santiago, Chile; ^4^Millennium Nucleus of Ion Channel Associated Diseases (MiNICAD), Universidad de Chile, Santiago, Chile

**Keywords:** Neuropilin-1, tolerance, Treg cells, immune regulation, transplantation

## Abstract

Several mechanisms of immune suppression have been attributed to Foxp3+ T regulatory cells (Treg) including modulation of target cells via inhibition of cell proliferation, alteration of cytokine secretion, and modification of cell phenotype, among others. Neuropilin-1 (Nrp1), a co-receptor protein highly expressed on Treg cells has been involved in tolerance-mediated responses, driving tumor growth and transplant acceptance. Here, we extend our previous findings showing that, despite expressing Foxp3, *Nrp1KO* Treg cells have deficient suppressive function *in vitro* in a contact-independent manner. *In vivo*, the presence of Nrp1 on Treg cells is required for driving long-term transplant tolerance. Interestingly, Nrp1 expression on Treg cells was also necessary for conventional CD4+ T cells (convT) to become Nrp1+Eos+ T cells *in vivo*. Furthermore, adoptive transfer experiments showed that the disruption of Nrp1 expression on Treg cells not only reduced IL-10 production on Treg cells, but also increased the frequency of IFNγ+ Treg cells. Similarly, the presence of *Nrp1KO* Treg cells facilitated the occurrence of IFNγ+CD4+ T cells. Interestingly, we proved that *Nrp1KO* Treg cells are also defective in IL-10 production, which correlates with deficient Nrp1 upregulation by convT cells. Altogether, these findings demonstrate the direct role of Nrp1 on Treg cells during the induction of transplantation tolerance, impacting indirectly the phenotype and function of conventional CD4+ T cells.

## Introduction

Foxp3+ T regulatory (Treg) cells are an important population of leukocytes that control immunity, mainly by dampening effector T cell responses. Many studies have described the mechanisms by which Treg cells carry out their function, such as IL-2 deprivation, secretion of cytotoxic granules (granzyme/perforin), metabolic disruption, secretion of anti-inflammatory cytokines, and release of extracellular vesicles (exosomes) (1, 2).

In addition to their capacity to suppress immune responses, Treg cells had become an interesting target for cell therapy, due to the increasing number of diseases associated with malfunction and over-reactivity of the immune system, such as autoimmunity and transplant rejection (3, 4). The current paradigm is based on the premise that immune tolerance to allogeneic transplant is broken by an imbalance of Treg cells over T effector cells. The infusion of Treg cells considerably increases graft survival in transplanted animals (4, 5), and clinical trials of the administration of Treg cells into patients have demonstrated safety but variable efficacy (6). Understanding Treg cell biology and its mechanisms of immune suppression may improve the potential and use of Treg cells as therapeutic agents.

A few years ago, Neuropilin-1 (Nrp1) was described as a potential Treg cell marker. Nrp1 is a transmembrane co-receptor with affinity for a variety of ligands, all involved in physiological processes, such as angiogenesis, axonal guidance, or immune synapses (7). In the immune system, Nrp1 is expressed mainly by dendritic cells, Natural Killer (NK) and Treg cells (8–11). Initially, the function described for Nrp1 was to stabilize the interaction between cells during antigen presentation through homotypic interactions (12, 13). However, other studies suggested later that Nrp1 contributes to the function, phenotypic stability, and survival of Treg cells in tumors (14).

Several reports correlate Nrp1 expression on T cells with a state of immune tolerance (14–19), which has been demonstrated in the transplantation context both in patient biopsies and experimental models (20–22). In addition, Nrp1-deficient or *Nrp1KO* Treg cells are not capable of exerting suppressive function through a semi-porous membrane; and the same phenomenon was observed when using wild type Treg cells in the presence of anti-Nrp1 blocking antibodies (14).

We previously described that conventional CD4+ T cells (defined as CD4+CD25-Nrp1-Foxp3-cells or convT) up-regulate Nrp1 expression during allograft rejection. Interestingly, in the tolerogenic condition in which Nrp1+Foxp3+ Treg cells are co-transferred with convT cells, a larger frequency of Nrp1+Eos+ convT cells was observed suggesting that Nrp1+Treg cells could modulate the phenotypic signature of convT cells (22), leading to the generation of T cells with modulatory effects.

Based on these antecedents, we hypothesized that convT cells gain Nrp1 and Eos in an Nrp1+Treg cell-dependent manner to favor immune suppression. Using Nrp1 conditional knocked out mice; we demonstrate that *Nrp1KO* Treg cells are deficient in exerting suppressive activity in a contact-independent manner. Even more, when Treg cells lack Nrp1, convT cells are unable to up-regulate Nrp1 and Eos expression favoring the appearance of type-1 T helper (Th1) cells. Accordingly, the frequency of IL-10+Treg cells is negatively affected, which correlates with the inability to induce long-term tolerance. Lastly, we demonstrate that Treg cells-modulated convT cells also gain the ability to suppress *ex vivo* T cell proliferation, which is affected if co-transferred Treg cells lack Nrp1. Hence, we demonstrate that Treg cells drive immune tolerance by modulating the phenotype and function of convT cells in an Nrp1-dependent manner.

## Results

### The Lack of Nrp1 on Treg Cells Is Not Involved in Treg-Phenotypic Signature

**Figure 1:**
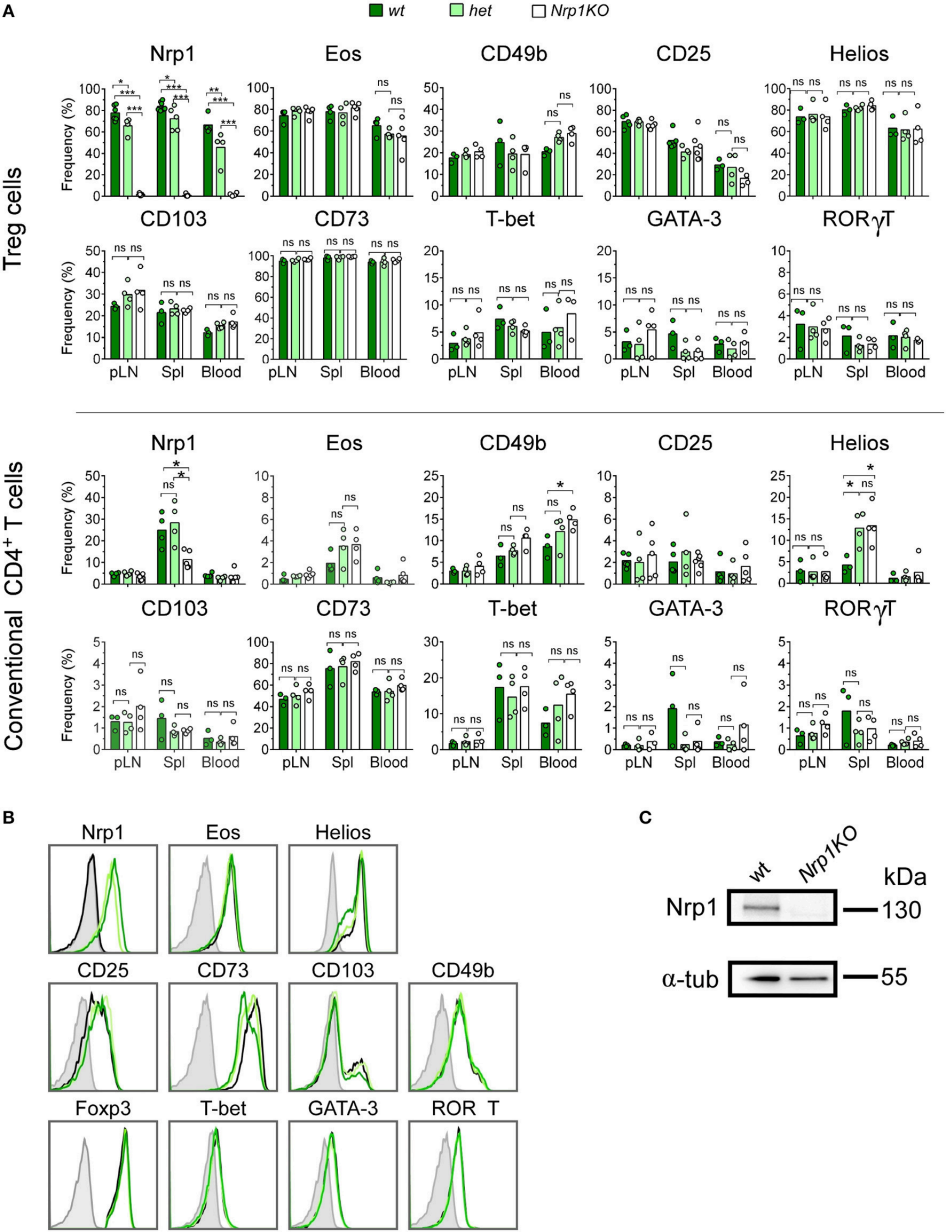
Foxp3-targeted Nrp1 deletion only occurs on Treg cells and does not affect conventional T cell phenotype. Peripheral lymph nodes (pLN), spleen (Spl), and blood were harvested from Foxp3^YFP/Cre^Nrp1^+/+^ (*wt*, dark green bars), Foxp3^YFP/Cre^Nrp1^+/flox^ (*het*, light green bars), and Foxp3^YFP/Cre^Nrp1^flox/flox^ (*Nrp1KO*, white bars) animals. Leukocytes were obtained and tested for the indicated markers by flow cytometry. **(A)** Graphs show the frequency of Nrp1, Eos, CD49b, and CD25 on Foxp3+ Treg cells and conventional CD4+T (convT) cells from *wt, het*, and *Nrp1KO* Treg cell-restricted mice. **(B)** Representative histograms showing the expression of indicated markers on Foxp3+ Treg cells from *wt* (dark green line), *het* (light green line), *Nrp1KO* (black line) mice; and FMO control (gray line). **(C)** Immunoblot of Nrp1 protein expression on *wt* and *Nrp1KO* Treg cells. For A, *n* = 3–4 animals per genotype, each circle represents one mouse, bars represent mean. Two-way ANOVA, ^*^ < 0.05; ^**^ < 0.01; ^***^ < 0.001; ns, not significant. Representative data of three independent experiments.

In 2015, our group reported that convT cells transferred into skin-transplanted animals gain Nrp1 expression (from 0 to ~35%). This induction was highly increased when convT cells were co-transferred with Nrp1+Treg cells (22). To clarify whether this process depends on Nrp1 expressed specifically on Treg cells, we obtained Foxp3^Cre/YFP^ and Nrp1^flox/flox^ mice to generate Nrp1-deficient or *Nrp1KO* Treg cells, which are conveniently detectable by flow cytometry based on the expression of YFP (23, 24). First, we tested the phenotype of T cells from different organs/tissue of *Foxp3*^Cre/YFP^ (wild type, *wt* control), *Foxp3*^Cre/YFP^*Nrp1*^flox/+^ (*het*) and *Foxp3*^Cre/YFP^ *Nrp1*^flox/flox^ (*Nrp1KO* Treg) offspring. As expected, we found that deletion of Nrp1 only occurs on Treg cells, as seen in peripheral lymph nodes (pLN), spleen (Spl), and blood cells (~75% on *wt* and *het*, and < 1% on *Nrp1KO*) (Figure 1A). In the case of convT cells, we find a partial decrease in Nrp1 expression only in the Spl, which could correspond to antigen experienced ex-Treg cells (Foxp3-T cells), since convT cells were considered and gated as Foxp3-cells.

In addition, CD8+ T cells express very low levels of Nrp1 (< 5%), which did not change either in *het* or *Nrp1KO* Treg cells (Supplemental Figure 1A) (15). We also analyzed the expression of Treg cell-associated markers such as Eos (25, 26), CD49b (27), CD25 (28), Helios (23), CD103 (29–31), and CD73 (32, 33) along with T-bet, GATA-3, and RORγT as lineage transcription factors for T helper (Th)-1, Th2, and Th17 cell subsets, respectively (34–36) on convT and Treg cells observing no differences among all three genotypes (Figures 1A,B). The absence of Nrp1 on splenic Treg cells obtained from *Nrp1KO*-animals was corroborated using western blot assay (Figure 1C).

**Figure 2:**
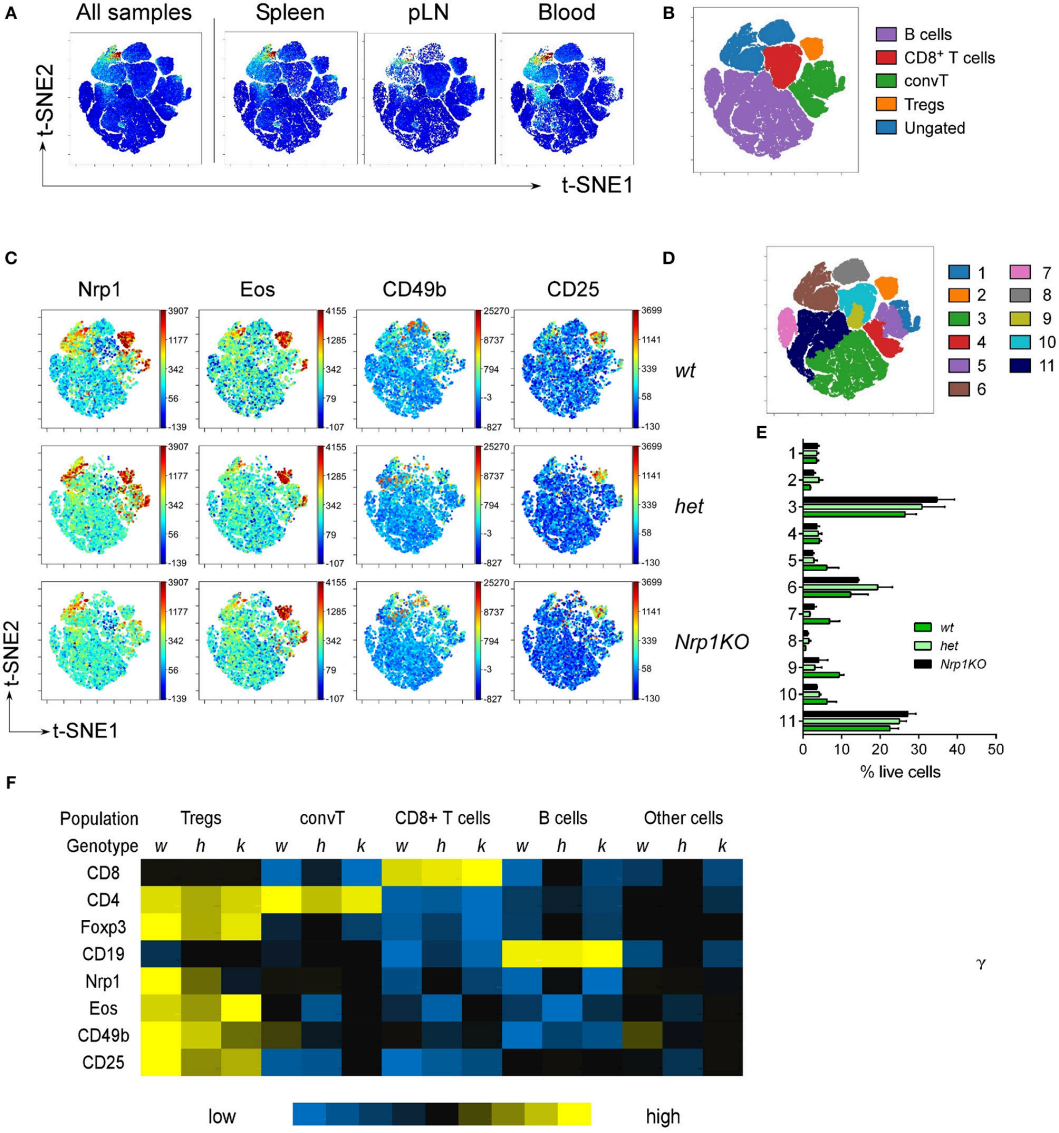
Disruption of Nrp1 expression on Treg cells does not interfere with the acquisition of modulatory molecules. Organs and tissues were collected and processed as in Figure 1. viSNE analysis was run on 10,000 live single cells per sample using cell subtype markers. **(A)** Representative viSNE map of concatenated files of all samples, and concatenated individual populations from pLN, Spl, and blood samples. **(B)** viSNE map showing color-coded cell populations based on lineage marker expression gating. **(C)** Fluorescence intensity for specific markers from *wt, het*, and *Nrp1KO* spleen cells on viSNE map. **(D)** SPADE-on-viSNE map defining 11 spatially distinct splenic cell populations. **(E)** Relative proportions of immune cell populations defined in **(D)** from splenocytes. **(F)** MFI heat map of surface and intracellular markers expression on each immune cell subpopulation from splenic Treg-restricted *wt* (*w*), *het* (*h*), and *Nrp1KO* (*k*) mice. Representative data of at least three independent experiments.

Even more, we performed high-dimensional single cell data analysis by visualization of t-Distributed Stochastic Neighbor Embedding (t-SNE) algorithm (viSNE) (37, 38) on pLN, Spl and blood cells populations from all three mice genotypes (Figures 2A,B), searching for main cell subsets based on the expression of CD8, CD4, Foxp3, and CD19 (Supplemental Figure 1B). viSNE heat maps confirmed that Nrp1 depletion only occurs in the Foxp3+Treg cell compartment of *Nrp1KO* animals, whereas expression of Nrp1, Eos, CD49b, and CD25 remain mostly unchanged among the identified populations (CD19+ B cells, CD4+Foxp3-convT, and CD8+ T cells) (Figures 2B,C). Additionally, by performing spanning-tree progression analysis of density-normalized events (SPADE), we identified 11 spatially distinct immune cell populations with unaltered frequencies among *wt, het*, and *Nrp1KO* splenic cells (Figures 2D,E). Furthermore, the relative Mean Fluorescence Intensity (MFI) of each marker was calculated for each SPADE-on-viSNE cell population studied, highlighting the lack of Nrp1 expression on *Nrp1KO* Treg cells among different tissues (Figure 2F).

### Treg Cells Require Nrp1 Expression to Modulate Conventional CD4+ T Cell Phenotypic Signature

**Figure 3:**
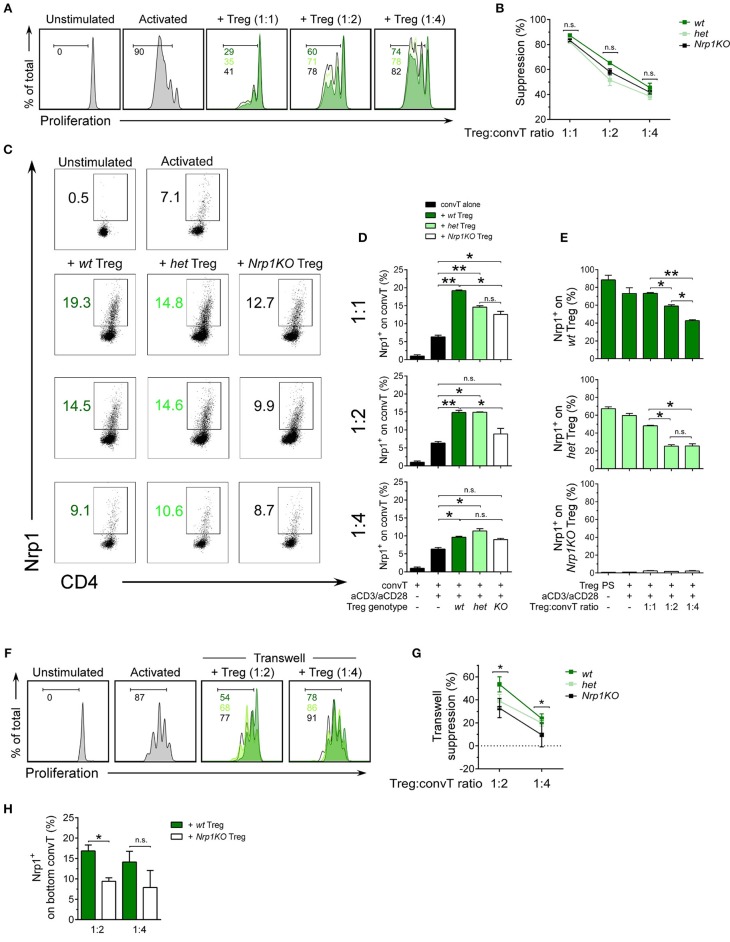
Nrp1 is required for immune-cell suppression and modulation of conventional CD4+ T phenotype. Responder convT cells were sorted, CTV-labeled and cultured with Mitomycin-C treated–APCs plus soluble anti-CD3 antibody, alone or in the presence of sorted Treg cells. **(A)** Histograms showing proliferation of convT cells activated alone (gray) or at different Treg:convT ratios (1:1, 1:2, and 1:4) of *wt* (dark green), *het* (light green), or *Nrp1KO* (black) Treg cells. **(B)** Percentage of suppression activity was calculated as described in Materials and Methods. Representative data of at least three independent experiments. Squares represent mean ± SEM. **(C)** Representative dot plots showing Nrp1 expression on convT cells activated as in **(A)**. **(D)** Pooled data depicting Nrp1 expression on CD45.1^+^convT cells cultured with Treg cells from the three indicated genotypes. Bars represent mean ± SEM. Data is representative of at least two independent experiments. **(E)** Pooled data depicting Nrp1 expression on CD45.2^+^ Treg cells before and after co-culture with convT cells at aforementioned different Treg:convT ratios. PS: post-*sorting* expression of Nrp1 on Treg cells. **(F)** Histograms showing proliferation of convT cells activated alone (gray) of at different Treg:convT ratios (1:2 and 1:4) of *wt* (dark green), *het* (light green), or *Nrp1KO* (black) Treg cells from transwell experiments (described in detail in Materials and Methods section and Supplemental Figure 3). **(G)** Percentage of suppression activity was calculated as described in Materials and Methods. Representative data of two independent experiments performed in triplicates. **(H)** Pooled data depicting Nrp1 expression on CD45.1^+^ convT cells cultured with *wt* or *Nrp1KO* Treg from transwell experiments. Bars represent mean ± SEM. Data is representative of two independent experiments performed in triplicates. For **(B,D–E,H)**, Paired *T*-test; ^*^ < 0.05; ^**^ < 0.01 were used; ns, not significant. For **(G)** ^*^ < 0.05 for *wt* vs. *Nrp1KO* (both 1:2 and 1:4 ratios).

After confirming typical Treg cell phenotype in all genotypes, we performed a suppression assay to test the modulatory function of Treg cells and acquisition of Nrp1 by convT cells *in vitro*. For this experiment we used congenically marked antigen presenting cells (APC, CD45.2+), Treg cells (CD45.2+) and CTV-stained convT cells (CD45.1+) to facilitate the tracking of convT cells under proliferation (Supplemental Figure 2A). As shown in Figure 3A, *wt* Treg cells suppress the proliferation of convT cells at 1:1, 1:2, and 1:4 Treg:convT ratios (~85, ~65, and ~45% of suppression, respectively); but both *het* and *Nrp1KO* Treg cells show slightly reduced suppressive activity, although these variations are not statistically different. These results suggest that Nrp1 is not fully required for Treg cells acting in a contact-dependent manner (Figure 3B). On the other hand, we checked the phenotype of convT cells by looking at Nrp1 expression. As depicted in Figures 3C,D, convT cells acquired Nrp1 expression in a dose and Treg-dependent manner as *Nrp1KO* Treg cells were able to allow convT cells to become Nrp1+ to a lesser extent (for instance, at 1:1 ratio we observe ~5% of Nrp1+ conv T cells without Treg cells, ~20% of Nrp1+ convT cells with *wt* Treg cells, ~15% of Nrp1+ convT cells with *het* Treg cells and ~12% of Nrp1+ convT cells with *Nrp1KO* Treg cells). This data supports our previous observation, in which convT cells up-regulated Nrp1 during allograft rejection but when convT cells were co-transferred with Treg cells, the expression of Nrp1 was enhanced in addition to the induction of transplant tolerance (22). At the same time, Nrp1-expressing Treg cells (*wt* and *het*) decrease Nrp1 expression in a dose-dependent manner when co-cultured with convT cells at the different ratios, Figure 3E. Conversely, convT cells co-cultured with *wt* Treg cells did not gain Eos expression *in vitro*, which could be a result of the experimental timing (3 days for *in vitro* assay vs. 20 days for *in vivo* assay) (Supplemental Figures 2B,C).

Since we did not observe a robust difference between the suppressive activity of *wt, het*, and *Nrp1KO* Treg cells, we repeated the suppression assay but using a contact-independent setting (transwell system) (Supplemental Figure 3). In this case, we observed differences in the suppressive function of *wt* and *Nrp1KO* Treg cells, where *Nrp1KO* Treg show reduced suppressive activity, indicating that Nrp1+ Treg cells produce factors with modulatory function, which are altered in *Nrp1KO* Treg cells, Figures 3F,G. Even more, convT cells also up-regulated Nrp1 expression when *wt* Treg cells, and not *Nrp1KO* Treg, were placed at the top chamber (~17% vs. ~9% of Nrp1+ convT cells, respectively, Figure 3H). Thus, the presence of Nrp1 on Treg cells modulates the phenotype of convT cells and contributes to T cell suppression *in vitro*.

### Nrp1KO Treg Cells Cannot Induce Long-Term Transplant Tolerance and Fail to Modulate Conventional CD4+ T Cells *in vivo*

**Figure 4:**
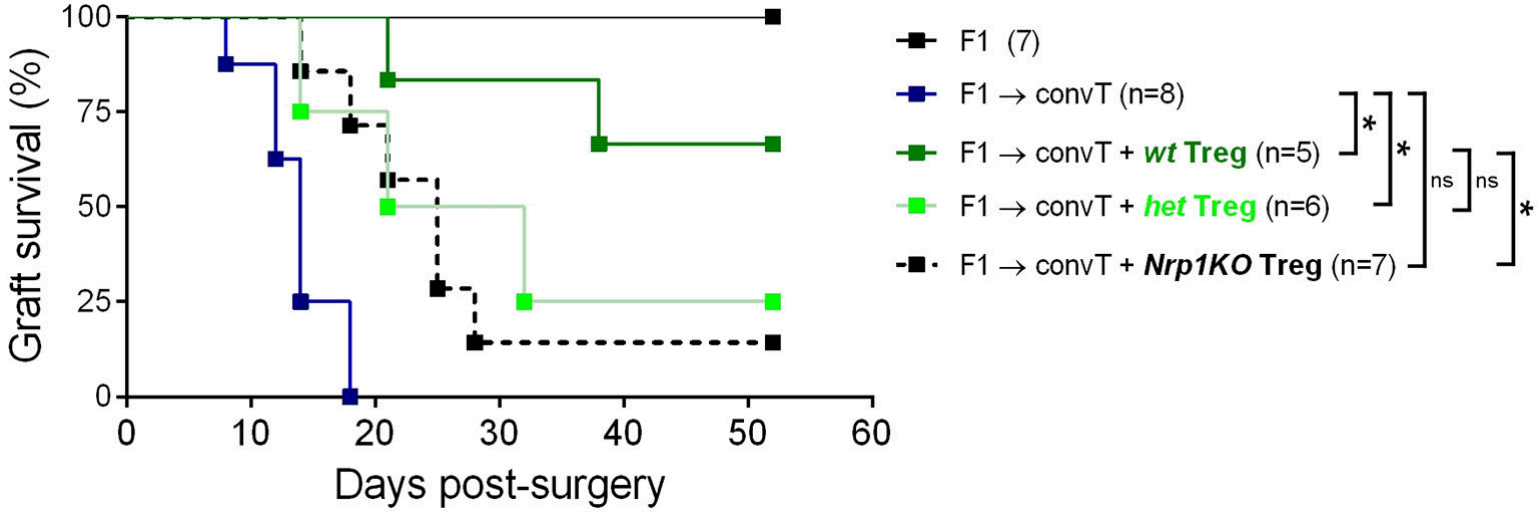
*Nrp1KO* Treg cells are unable to exert long-term skin transplantation tolerance. Conventional CD4+ T cells were sorted and adoptively transferred alone or with sorted Foxp3/YFP+CD45.2+ Treg cells into RAG-KO recipient mice. Next day, mice were transplanted with F1 semi-allogeneic skin grafts (day 0) and graft survival was monitored over time. Long-term skin allograft survival was compared between mice receiving *wt* (dark green), *het* (light green), or *Nrp1KO* (black) Treg cells. Lines represent graft survival (%), log-Rank test: ^*^p < 0.05 between “convT cells” and “*wt*”; ^*^p < 0.05 between “convT cells” and “*het*”; ^*^p > 0.05 between “convT cells” and “*Nrp1KO*”; ^*^p > 0.05 between “*wt*” and “*het*”; ^*^p < 0.05 between “*wt*”and “*Nrp1KO*”.

Next, we tested the role of Nrp1 on Treg cell function using an *in vivo* skin transplantation model, in which C57BL/6 × Balb/c (F1) skin is grafted onto RAG-KO mice previously administered with convT cells (Supplemental Figure 4A). Using this approach, we observed complete transplant rejection by day 20 post-surgery, but skin graft tolerance when convT cells are co-transferred with Nrp1+ Treg cells (22). In the current study, we carried out long-term skin transplant experiments using *wt, het*, and *Nrp1KO* Treg cells and found that *wt* Treg cells induce ~60% of transplant survival and only ~20% for the groups receiving *het* and *Nrp1KO* Treg cells (Figure 4).

**Figure 5:**
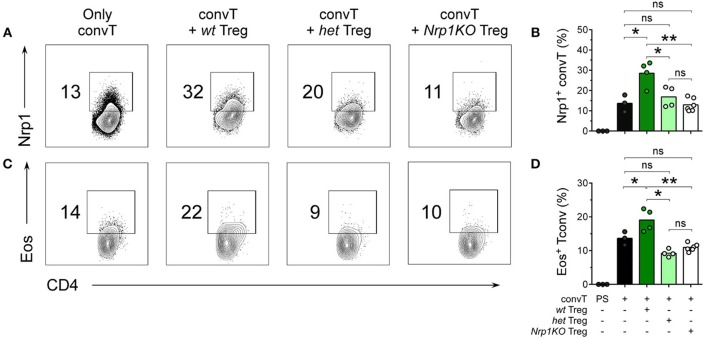
Nrp1+Treg cells are required for the acquisition of Nrp1 and Eos expression by conventional CD4+ T cells during skin transplantation acceptance. Mice were transplanted as described in Figure 4. At day 20 post-surgery, graft-draining lymph nodes were harvested and cells were analyzed for Nrp1 and Eos expression by flow cytometry. Representative contour plots depicting **(A,B)** Nrp1 and **(C,D)** Eos expression on gated live CD4+CD45.1+ convT cells. **(B–D)**, bars represent mean and each circle represents one mouse. Unpaired *T*-test, ^*^ < 0.05; ^**^ < 0.01; ns, not significant; PS, post sorting. Representative data of at least two independent experiments.

Furthermore, we performed 20 days-long experiments, for which skin graft-draining lymph nodes (dLN) were removed and the number and phenotype of T cells was studied. All three Treg cell genotypes were able to reduce cellularity in the dLN of allografted animals (Supplemental Figure 4B). Additionally, we analyzed dLN cell phenotype by flow cytometry, discriminating clearly between convT cells (CD45.1+) and Treg cells (CD45.2+) (Supplemental Figures 4C,D). As depicted in Figures 5A,C, convT cells gained Nrp1 and Eos expression, which seems to occur in a Treg cell-dependent manner because ~30% of convT cells became Nrp1+ when co-transferred with *wt* Treg cells in comparison with ~15% or ~12% when co-transferred with *het* or with *Nrp1KO* Treg cells, respectively, Figure 5B. Moreover, ~20% of convT cells gained Eos expression when co-transferred with *wt* Treg cells in contrast to ~10% for the case of *het* or *Nrp1KO* Treg cells, Figure 5D. Importantly, Eos expression remain unaltered regardless of Treg cells genotype, Supplemental Figures 4D–G. These observations confirmed our previous study in which we described that CD4+ T effector phenotype is modulated by Nrp1-expressing Treg cells.

**Figure 6:**
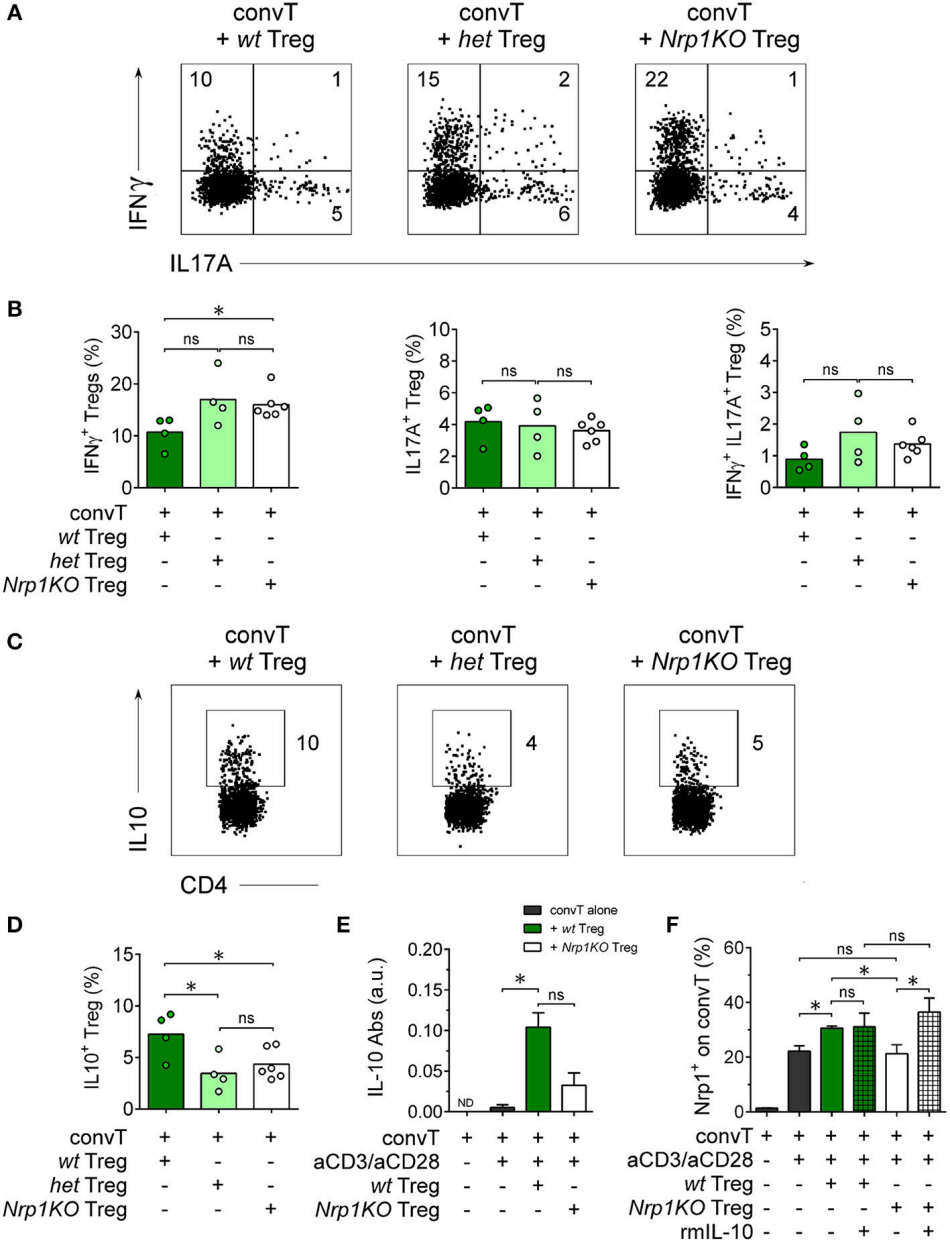
Nrp1 controls IFNγ and IL-10 production by Treg cells. Mice were transplanted as described in Figure 4 and cells processed as in Figure 5. Representative dot plots **(A)** and bar graphs **(B)** showing the expression of IFNγ and IL17 on *wt* (dark green), *het* (light green), or *Nrp1KO* (white) Treg cells. Representative dot plots **(C)** and bar graphs **(D)** depicting the expression of IL-10 on *wt, het*, or *Nrp1KO* Treg cells. **(E)** Responder convT cells and Foxp3^+^ Treg cells were sorted and treated as described in Figure 3. After 72 h, secreted IL-10 was measured in the supernatant of convT cells cultured alone or with Treg from *wt* or *Nrp1KO* mice by ELISA. **(F)** Pooled data depicting Nrp1 expression on CD45.1^+^ convT cells cultured with Treg cells from the two indicated genotypes, alone or plus recombinant murine IL-10 (rmIL-10). For **(B,C)**, bars represent mean and each circle represents one mouse. Unpaired *T*-test, ^*^ < 0.05; ns, not significant. Pooled data of at least two independent experiments. For **(E,F)**, bars represent mean ± SEM. Paired *t*-Test, ^*^ < 0.05; ns, not significant. Pooled data of two independent experiments performed in triplicates.

To further investigate *Nrp1KO* Treg cells, we studied Treg cell cytokine production finding that ~20% of either *het* or *Nrp1KO* Treg cells were IFNγ+ in contrast to~10% of *wt* IFNγ+ Treg cells, Figures 6A,B. No differences were observed among the frequencies of *wt, het*, or *Nrp1KO* Treg cells either IL-17A+ or IFNγ+IL-17A+. When we tested for the production of the anti-inflammatory cytokine IL-10, we found that < 5% of *het* and *Nrp1KO* Treg cells were IL-10+ compared with ~8.5% of *wt* Treg cells, Figures 6C,D. Therefore, Nrp1-deficiency on Treg cells during allograft rejection negatively affects IL-10 production but favors IFNγ production, which may explain the reduced % of transplant survival of grafted animals treated with *Nrp1KO* Treg cells. To corroborate this, we analyzed IL-10 levels on convT-and-Treg *in vitro* culture supernatants, observing decreased IL-10 production when convT were co-cultured with *Nrp1KO* Treg cells, compared with *wt* Treg (Figure 6E). According to our previous results, convT cells upregulate Nrp1 expression when cultured with *wt* Treg but not with *Nrp1KO* Treg cells (~30% Nrp1+ convT vs. ~20% Nrp1+ convT cells cultured with *Nrp1KO* Treg or alone, Figure 6F). Notably, the addition of exogenous IL-10 to the culture appeared to rescue the gaining of Nrp1 expression by convT cells supporting the notion that decreased IL-10 production contributes to the impaired suppressive function of *Nrp1KO* Treg and their immune modulation on convT cells.

**Figure 7:**
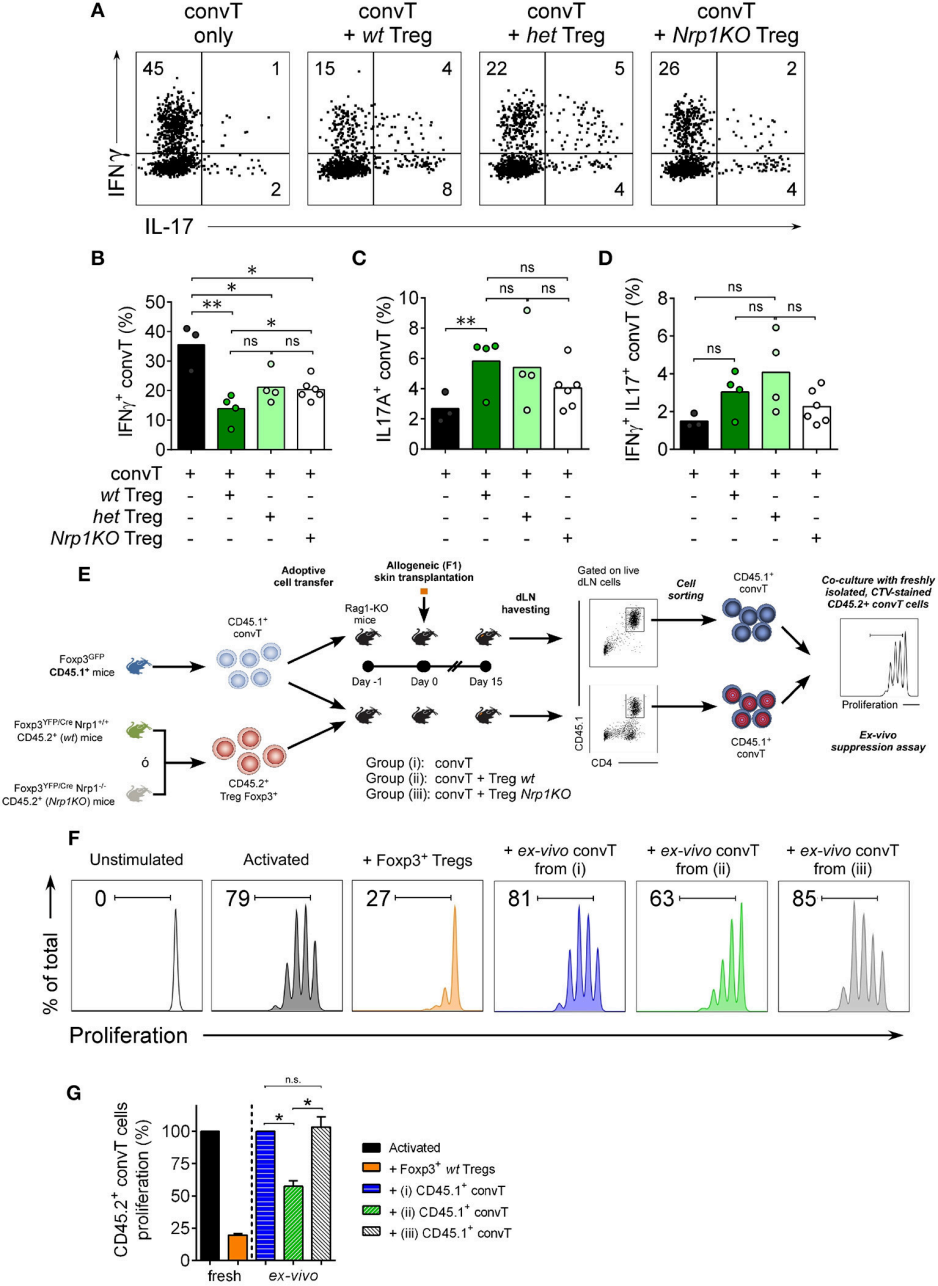
Nrp1+Treg cells modulate conventional T cell phenotype and function. Mice were transplanted as described in Figure 4. At day 20 post-surgery, graft draining lymph node cells were obtained and stimulated with PMA and Ionomycin in the presence of Brefeldin A. Next, intracellular cytokine staining was performed and the expression of IFNγ and IL-17 on convT cells was determined by flow cytometry. **(A)** Representative dot plots showing the expression of IFNγ and IL-17 on CD4+CD45.1+ convT cells when transferred alone or with Treg cells from *wt, het*, or *Nrp1KO* Treg cells. Bar graphs depicting the frequencies of **(B)** IFNγ+CD4+ T, **(C)** IL-17+CD4+ T, and **(D)** IFNγ+IL-17+CD4+ convT cells when transferred alone (black) or with *wt* (dark green), *het* (light green), or *Nrp1KO* (white) Treg cells. Pooled data from two independent experiments, each circle corresponds to one mouse. Unpaired *T*-test, ^*^ < 0.05; ^**^ < 0.01; ns, not significant. **(E)** Mice were transplanted as described in Figure 4. At day 15 post-surgery, graft draining lymph node cells were obtained, stained and live CD45.1+CD4+ convT cells were FACS-sorted and tested for suppression function over freshly isolated, CTV-stained CD45.2+ CD4+ convT cells activated with MHC-II+ APCs and soluble anti-CD3 for 72 h. **(F)** Histograms showing proliferation of fresh CD45.2+ CD4+ convT cells activated alone (dark gray) or in the presence of control Foxp3+ wt Treg (orange) or CD45.1+CD4+ convT cells of dLN of RAG-KO allografted mice from Group (i) (convT alone, blue), Group (ii) (convT plus *wt* Treg cells, green), or Group (iii) (convT plus *Nrp1KO* Treg cells, light gray). **(G)** Percentage of proliferation of fresh CD45.2+ CD4+ convT cells was calculated as described in Materials and Methods. Representative of at least three independent experiments. Bars represent mean ± SEM. Paired *T*-test, ^*^ < 0.05 was used.

When we analyze the convT cell subset, we found ~40% of IFNγ+CD4+ convT cells in the rejecting group; this frequency was significantly reduced to ~15% in the presence of *wt* Treg cells, but only to ~25% of convT cells in the presence of *het* or *Nrp1KO* Treg cells, Figures 7A,B. The frequencies of IL-17+CD4+ T and IFNγ+IL-17+ CD4+ T cells were not changed in the presence of *Nrp1KO* Treg cells, Figures 7C,D. Strengthening this observation, and the fact that *wt* Treg cells modulate convT cells phenotype, we sorted out convT cells from transplanted animals that received either *wt* or *Nrp1KO* Treg cells, and tested them for *in vitro* inhibitory function (Figure 7E), finding that the former also renders suppressive function, as shown in the *ex vivo* suppression assay (~55% proliferation of freshly isolated CD45.2+ convT cells co-cultured with “*wt* Treg-modulated CD45.1+ convT cells” vs. freshly isolated convT cells co-cultured with effector T cells from rejecting animals), Figures 7F,G. Accordingly, convT cells become less suppressive when Treg cells are defective in Nrp1 expression (~105% proliferation of fresh CD45.2+ convT cells co-cultured with “*Nrp1KO* Treg-modulated convT cells”).

Altogether, our findings indicate that Treg cells require Nrp1 to modulate the function of convT cell and to exert optimal suppressive activity *in vivo*.

## Discussion

Regulatory T cell malfunction has been widely associated with increased inflammatory immune responses. Understanding Treg cells biological processes and mechanisms of suppression are pivotal for recognizing new targets for therapy. Nrp1 has been previously proposed as a cell marker for thymic-derived Treg cells (39); but its expression has also been described on T cells during allogeneic skin graft rejection (22), sepsis (40), IL-10 deficiency (41), and anti-tumor immune responses (42, 43). Moreover, Nrp1 deficiency on Foxp3+ Treg cells has been associated with lack of immune suppression, predominantly affecting tumor growth (14, 15, 44) and worsening EAE severity (45). In this work, we focused on analyzing the role of Nrp1 specifically on Treg cells during the induction of skin transplant tolerance. In one of our previous studies we demonstrated that Nrp1+ Treg cells drive transplantation tolerance, proposing the modulation of conventional CD4+ T cell phenotype as a possible mechanism (22). To get more insight regarding this observation, we obtained conditional knockout animals in which the lack of Nrp1 expression is restricted to Foxp3+ Treg cells (24). First, we extensively analyzed the phenotype of T cells in these genetically modified mice, including Foxp3+ Treg cells, finding no aberrant expression of Treg cell-associated markers in animals containing *Nrp1KO* Treg cells compared to controls (Figures 1, 2). Next, we designed *in vitro* experiments to evaluate the function of *Nrp1KO* Foxp3+ Treg cells and their capacity to modulate convT cell phenotype. The regulatory ability of *Nrp1KO* Foxp3+ Treg cells was evaluated in contact-dependent and—independent assays, observing that Foxp3+ Treg cells require Nrp1 to inhibit T cell proliferation in a contact-independent manner, suggesting that Nrp1+ Treg cells secrete modulators to exert their suppressive function (Figure 3). Because we obtained a defect in cell contact-independent suppression when using *Nrp1KO* Treg cells, and Delgoffe et al. reported that the inclusion of anti-Nrp1 blocking antibody in a transwell suppression assay negatively affected the suppressive capacity of Nrp1+ Treg cells (14), it is reasonable to propose that Nrp1+Foxp3+ Treg cells may secrete Nrp1+ extracellular vesicles (EV) to “deliver” Treg cells' modulatory effects to convT cells. In this regard, several authors have published that Treg cells are able to produce EV (containing modulatory molecules) as a novel manner to inhibit CD4+ T cell effector function (46–48). Furthermore, we also observed in the *in vitro* experiments that convT cells gained Nrp1 in a dose dependent manner according to the ratios used with Nrp1+ Treg cells (Figures 3C,D). In other words, we obtained higher % of Nrp1+ convT cells when co-cultured with higher number of Nrp1+ Treg cells, resulting in lower frequencies of Nrp1+ convT cells when *Nrp1KO* Treg cells were added. Even more, Nrp1+ Treg cells decreased Nrp1 expression while convT cells gained it (Figures 3D,E). This phenomenon was first reported by our group using an *in vivo* model of transplantation and in this current report we corroborated the observation *in vitro* in addition to proving that it occurs in Nrp1+Foxp3+ Treg cell-dependent manner (Figure 3). Hence, it is conceivable to propose that Nrp1+ Treg cells could secrete Nrp1+ EV to target convT cells; in this regard, data from our laboratory indicates that *wt* Treg cells secrete EV containing Nrp1 in their membrane and that these EV can modulate convT cells phenotype and function, in comparison to EV obtained from *Nrp1KO* Treg cells (manuscript in preparation).

Using a murine model for allograft transplantation, we demonstrated that Nrp1 is required by Foxp3+ Treg cells to facilitate long-term tolerance (Figure 4). Interestingly, when we studied the phenotype of *wt* and *Nrp1KO* Treg cells in this *in vivo* setting, we did not find changes in Foxp3 or Eos expression (Supplemental Figures 4D–F). The transcription factor Eos functions as a co-repressor of Foxp3, preventing the expression of convT cell-related genes in Treg cells (26); and it has been described that “Eos-labile” Treg cells display a more inflammatory/helper phenotype (25). On the contrary, Rieder et al. have reported that convT cells from Eos^−/−^ animals produce less IL-2 and show a malfunctioning CD25/STAT5 signaling pathway upon *in vitro* activation, but under *in vivo* inflammatory conditions Eos^−/−^T cells become high producer of IL-17 (49). In this work, we found that Nrp1 deficiency did not modify Foxp3 or Eos expression on Foxp3+ Treg cells under homeostatic conditions and in our model of skin graft transplantation, supporting that graft rejection was not due to instability in the expression of Foxp3 or Eos. Complementing Treg cell phenotypic analysis, we found that *Nrp1KO* Treg cells up-regulate IFNγ (Figure 6), supporting that the Nrp1 signaling pathway is required by Treg cells to secrete the appropriate cytokines (44). Gao et al. showed that Nrp1^low^ CD25+ CD4+ Treg (Treg expressing low levels of Nrp1) purified from septic mice secreted lower amounts of IL-10 (17), and another study using tumor-harboring *Il10KO* (IL-10 deficient)-mice showed decreased Nrp1 expression in tumor-infiltrating Treg, impaired Nrp1+ Treg tumor accumulation and tumor protection function (41). While RNA sequence observations by Delgoffe et al. previously showed decreased *Il10* expression on *Nrp1KO* Treg (14), we have demonstrated here, for the first time, an impairment in IL10 production [both *in vitro* (in co-culture suppression assay) and *in vivo* (during allograft rejection), Figure 6] on Treg cells lacking Nrp1 expression. The gaining of Nrp1 expression by convT cells failed in the presence of *Nrp1KO* Treg (Figure 3) but, intriguingly, it could be reversed by adding exogenous IL-10 to the culture media, suggesting that Treg-mediated upregulation of Nrp1 on convT cells (and possibly other immunomodulatory proteins) could be dependent on IL-10 signaling.

On the other side, convT cells gained Nrp1 and Eos expression *in vivo* when co-transferred with *wt* Treg cells, which is in agreement with our previous findings (22). Notably, both *het* and *Nrp1KO* Treg cells are unable to favor Nrp1 and Eos expression on convT cells *in vivo*, and T cell suppression activity *ex vivo*, which correlates with increased IFNγ+ convT cell frequencies and poor allograft acceptance observed (Figures 4, 5, 7). These data support the hypothesis involving the secretion of Nrp1+ EV by Treg cells to modulate the phenotype/function of convT cells and the role of Nrp1 in controlling cytokine production of CD4+ T cells, although this control will depend on the subset of CD4+ T cells targeted (Treg vs. convT cells).

Altogether, our current results indicate that the expression of Nrp1 on Foxp3+ Treg cells is relevant for driving T cell suppression *in vitro* and *in vivo* by modulating convT cell proliferation, phenotype, cytokine production, and suppressive function.

## Materials and Methods

### Mice

Six to 8 week old male and female mice were used. *Foxp3*^*Cre*−*YFP*^ (24), *Nrp1*^*flox*/*flox*^ (50), and RAG-KO mice were purchased from The Jackson Laboratory (Maine, USA). BALB/c (H-2^d^), C57Bl/6 (H-2^b^), F1 (C57Bl/6 × BALB/c, H-2^b/d^), and *Foxp3*^GFP^ (CD45.1 + or Ly5.2) (51) mice were maintained under pathogen-free conditions at the animal facility located at Facultad de Medicina, Universidad de los Andes. All procedures were reviewed, approved and carried out according to the bioethics committee guidelines from Universidad de los Andes, and National Commission of Science and Technology (CONICYT).

### Flow Cytometry

After cell viability staining with ZombieDye NIR (Biolegend, CA, USA), cell suspensions were stained in 1X PBS with 5% heat-inactivated fetal bovine serum (Gibco, USA) with the following antibodies anti-: CD3e (clone 145-2c11), CD4 (clone RM4-5), CD8 (clone 53-6.7), CD19 (clone 6D5), CD25 (clone PC61), CD45.1 (clone A20), CD45.2 (clone 104), I-A/I-E (clone M5/114.15.2), CD103 (clone 2E7), CD49b (clone Hma2), CD73 (clone TY/11.8), Nrp1 (clone 3E12), all from Biolegend, CA, USA. Surface staining was performed for 30 min at 4°C. Intracellular staining was performed using anti-: Helios (clone 2-F6, Biolegend), GATA-3 (clone 16E10A23, Biolegend), Eos (clone ESB7C2, ThermoFisher Scientific), Foxp3 (clone FJK-16s), RORγt (clone B2D), and the Fixation/Permeabilization Staining Kit (all from eBioscience, CA, USA), following manufacturer's instructions. For cytokine expression analysis, cells were activated with 50 ng/mL PMA (Sigma) and 1 μg/mL Ionomycin (Sigma) in RPMI containing 10% FBS and Brefeldin-A (eBioscience, CA, USA) for 4 h. Cells were stained for surface markers, fixed in Fixation Buffer and washed with the Intracellular Staining Permeabilization Wash Buffer (all from Biolegend, CA, USA). Intracellular cytokine staining was performed using anti-: IFNγ (clone XMG1.2), IL-17A (clone TC11-18H10.1), and IL-10 (clone JES5-16E3). Cells were sorted on a FACSAria II (BD Biosciences) and/or analyzed on a FACSCanto II (BD Biosciences, USA). Data analysis was performed using FlowJo software (CA, USA). To perform visualization of complex flow cytometry data, we used the Cytobank computational tool viSNE (visualization of t-Stochastic Neighbor Embedding) that generates a two-dimensional map in which cell distance represents the distance between cell parameters in high-dimensional space (37). Thus, cells that were phenotypically similar for the analyzed markers will be closer in a viSNE map (38, 52). To generate viSNE maps, samples were uploaded to Cytobank, live single cells were gated based on cell size and length and negative to Zombie Dye viability staining. Then, between 150,000 and 160,000 cell were subsampled from the data. After subsampling, viSNE was run at default parameters (1,000 iterations, random seed, perplexity = 30, theta = 0.5). viSNE maps were visualized using Cytobank interface, which was used to generate figures (color coding by marker expression levels).

### Skin Transplantation

Tail skin (~1 cm^2^) from C57Bl/6 (syngeneic) or F1 (allogeneic) donors was transplanted onto the dorsal area of RAG-KO recipient mice. Survival of skin allografts was evaluated twice per week and grafts were considered rejected when 80% of the initial graft had disappeared or become necrotic.

### Adoptive Transfer Experiments

Spleen and lymph node CD4+ T cells were pre-enriched using the CD4+ T Cell Isolation Kit, mouse (MiltenyiBiotec, CA, USA). CD4^+^Foxp3^GFP−^Nrp1^−^ cells were *sort-purified* as CD4+ T conventional cells from *Foxp3*^GFP^CD45.1^+^reporter mice, and CD4^+^Foxp3^Cre/YFP^ were FACS-sorted as Treg cells from *Foxp3*^Cre/YFP^CD45.2^+^ (*wt*), *Foxp3*^YFP−Cre^*Nrp1*^+/flox^CD45.2^+^ (*het*), or *Foxp3*^YFP−Cre^*Nrp1*^flox/flox^CD45.2^+^ (*Nrp1KO*) mice. CD4+ conventional T cells (1.5 × 10^5^) were intravenously (i.v) injected alone or co-transferred with Treg cells (5 × 10^4^) into RAG-KO mice, 1 day before transplant surgery. Unless otherwise stated, 20 days post-transplant mice were euthanized and graft draining lymph nodes (dLNs) were extracted for flow cytometry analysis.

### *In vitro* Suppression Assays

For contact-dependent assays, splenic CD4+CD25-Nrp1-Foxp3^GFP−^ cells were sorted from *Foxp3*^GFP^CD45.1+ mice as Responder T cells and labeled with 5 μM CellTrace™ Violet (CTV, ThermoFisher Scientific, MA, USA). CD3^−^MHC-II^+^ splenocytes were sorted from C57Bl/6 CD45.2^+^ mice and treated with 50 μg/mL of Mitomycin C (Calbiochem, MA, USA) to be used as antigen presenting cells (APC). Responder cells (5 × 10^4^) were polyclonally activated with 5 μg/mL anti-CD3 (clone 145-2c11, Biolegend) and Mitomycin-C (Mit-C) treated-APCs (1 × 10^5^), alone or co-cultured with different proportions of *wt, het*, or *Nrp1KO* Foxp3^YFP/Cre+^ Treg cells (1:1 to 1:4 Treg:TconvT ratio) in round-bottom, 96-well plates with 200 μL RPMI, 10% FBS, 1% HEPES (Gibco, USA), 1% Penicillin/Streptomycin solution (ThermoFisher, USA) (complete RPMI or cRPMI) for 72 h. In some experiments, recombinant murine IL-10 (rmIL-10, Peprotech, USA) was added at final concentration of 10 ng/mL. Responder T cell proliferation was analyzed by CTV dilution by flow cytometry using FACSCanto II (BD Biosciences). Suppression was calculated as previously described (53). In brief, it uses the formula % Suppression = (1 DI_Treg_/DI_Tresp_) × 100% (where DITreg stands for the division index of responder cells with Treg cells, and DITresp stands for the division index of responder cells activated without Tregs). For transwell suppression experiments, CTV-stained Responder cells (2.5 × 10^4^) were stimulated with anti-CD3 (2 μg/mL) and Mitomycin-C (5 × 10^4^) treated-APCs in 200 μL cRPMI in the bottom chamber of the multiwell plate, then 0.4 μm pore size transwell inserts (Corning, USA) were placed and *wt, het*, or *Nrp1KO* Tregs (1.25 × 10^4^) at different proportions (1:2 to 1:4 of Treg:convT cell ratio) activated with responder cells (1.25 × 10^4^) and APCs (2.5 × 10^4^) plus anti-CD3 in 100 μL on the top chamber for 72 h. Proliferation of responder CD45.2+ convT cells was analyzed as previously mentioned.

### *Ex vivo* Suppression Assay

CD45.1+ convT cells were sort-purified and i.v. injected into RAG-KO mice alone [“Group (i)”] or co-injected with CD45.2+Treg cells from *wt* or *Nrp1KO* animals [“Group (ii)” and “(iii),” respectively], followed by allograft transplantation the next day as aforementioned. Fifteen-days post-surgery, dLN were harvested and pooled from mice of the same group. Cells were stained for viability (ZombieDye NIR) and live CD45.1+CD4+ convT cells were FACS-sorted and co-cultured with freshly isolated CTV-stained CD45.2+ CD4+ convT cells. T cells were activated with Mit-C-treated MHC-II+ APC plus soluble anti-CD3 (at 1:1 ratio of fresh CD45.2+ convT:*ex-vivo* isolated CD45.1+ convT cells). After 72 h, the proliferation of CD45.2+ CD4+ convTcell was analyzed by CTV dilution using flow cytometry as previously mentioned. Proliferation was calculated using the following formula: CD45.2+ convT proliferation = [(DI_fresh+ex-vivo_)/(DI_fresh_)]^*^100, where DI_fresh+ex-vivo_ stands for division index of fresh CD45.2+ convT cells activated with control *wt* Tregs or *ex vivo* isolated CD45.1+ convT cells from RAG-KO animals, and DI_fresh_ stands for division index of fresh CD45.2+ convT cells activated alone.

### ELISA

Supernatants from co-culture assays were collected after 72 h and stored at −80°C until cytokine quantification by sandwich ELISA. Briefly, 96-well flat-bottomed plates were coated overnight with 1 μg/mL purified anti-mouse IL-10 (clone JES5-2A5, Biolegend) capture antibody. After several washing steps with 1X PBS + 0.05% Tween 20 and a blocking step with 1% BSA in 1X PBS, samples were incubated at room temperature for 2 h. Biotinylated anti-mouse IL-10 (clone JES5-16E3, Biolegend) at 1 μg/mL in conjunction with HRP-avidin (Biolegend) were used for detecting immobilized cytokine and tetramethylbenzidine (TMB, ThermoFisher) substrate was added to detect HRP activity. Reaction was stopped by adding 2N H_2_SO_4_ and absorbance was measured at 450 nm wavelength using a Tecan absorbance microplate reader.

### Western Blot

Inmunoblots were performed as previously described (54, 55). In brief, cell samples were lysed in RIPA buffer (10 mM Tris-HCl pH 6.8, 140 mM NaCl, 1 mM EDTA, 1% Triton X-100, 0.1% sodium deoxycholate, 0.1% SDS) on ice for 20 min. Lysates were vortexed and spinned at 17,200 × *g* for 20 min, and lysate supernatants were kept at −80°C until further use. The protein concentration was determined using Pierce BCA Protein Assay Kit (Life Technologies, US). 5X reducing sample buffer (60 mM Tris-HCl pH 6.8, 25% glycerol, 2% SDS, 5% β-mercaptoethanol and 0.04% bromophenol blue) was added to lysates at proper proportion, samples were heated at 95°C for 10 min and cooled to room temperature before resolved by SDS-PAGE (SDS-polyacrilamide gel electrophoresis). Proteins were resolved under fully denaturing and reducing conditions, transferred to nitrocellulose membrane, blocked in 4% non-fat powdered milk in PBS-T (0.1% Tween-20) and probed with antibodies. The following antibodies were used for Immunoblot: anti-α-Tubulin (clone B-7, Santa Cruz Biotechnologies, USA), and anti-Nrp1 (AF566, R&D Systems, USA), and peroxidase-coupled secondary antibodies anti-mouse IgG H+L (115-035-003, Jackson Immunoresearch, USA) and anti-goat IgG H+L (A27014, ThermoFisher, USA). Protein bands were detected using Pierce ECL Western Blotting Substrate (ThermoFisher, USA).

### Statistics

Statistical analyses were performed using Student's *t*-test, two-way ANOVA and log-Rank test using the software GraphPad Prism (CA, USA). Differences with *p* < 0.05 were considered significant. ^*^ < 0.05; ^**^ < 0.01; ^***^ < 0.001; ns, not significant.

## Ethics Statement

Mice were maintained under pathogen-free conditions at the animal facility located at Facultad de Medicina, Universidad de los Andes. All procedures were carried out according to the bioethics committee guidelines from Universidad de los Andes, and National Commission of Science and Technology (CONICYT).

## Author Contributions

MC-M performed experiments and analyze data. PC-K, FG-J, MR, CR, and AR participated in the execution of some experiments. OC discussed data and KP-L conceived and designed experiments, analyzed data, and wrote the manuscript.

### Conflict of Interest Statement

The authors declare that the research was conducted in the absence of any commercial or financial relationships that could be construed as a potential conflict of interest.

## Supplementary Material

Supplemental Figure 1 viSNE defines lymphocyte subsets in lymphoid tissues from wt, het, and Nrp1KO mice. Organs and tissue were collected as described in Figure 1. **(A)** Leukocytes were obtained from *wt* (dark green bars), *het* (light green bars), and *Nrp1KO* (white bars) animals and tested for the indicated molecules expressed by CD8+ T cells. **(B)** viSNE heat maps show the expression of CD8, CD4, Foxp3, and CD19 in order to distinguish CD8+ T cells, CD4+Foxp3—convT cells, CD4+Foxp3+ Treg cells and CD4-CD8-Foxp3-CD19+ B cells. For **(A)**, *n* = 3–4 animals per genotype, each circle represents one mouse, bars represent mean. Two-way ANOVA, ^**^ < 0.01; ^***^ < 0.001. For **(B)**, X-axis corresponds to t-SNE1 parameter, left Y-axis corresponds to t-SNE2 parameter and right Y-axis indicates MFI for each marker for all the viSNE heat maps. Representative data of three independent experiments.

Supplemental Figure 2 Expression of Eos on conventional T cells upon contact-dependent culture with Treg cells. **(A)** Contact-dependent suppressive assay strategy: Responder convT cells (CD4+CD25-Foxp3^GFP−^Nrp1-CD45.1+) were sort-purified from Foxp3^GFP+^CD45.1+ animals, antigen presenting cells (or APCs, CD3-MHCII+CD45.2+), and Treg cells (CD4+Foxp3^YFP+^CD45.2+) were sort-purified from *wt, het*, and *Nrp1KO*-Foxp3^YFP+^animals. ConvT cells were stained with CellTrace™Violet and cultured un-stimulated or activated with Mitomycin-C treated-APCs plus soluble anti-CD3 antibody, in the absence or presence of *wt, het*, or *Nrp1KO* Foxp3^YFP+^ Treg cells. ConvT cell proliferation was measured by dye dilution using flow cytometry. **(B)** Representative dot plots show Eos expression on CD45.1+convT cells after 3 days of co-culture with *wt* Treg cells. **(C)** Accumulated frequency of Eos+ convT cells in the aforementioned conditions. For C, bars represent mean ± SEM, *n* = 2 independent experiments.

Supplemental Figure 3 Contact-independent Treg cell suppression assay. Contact-independent suppressive assay strategy: responder convT, APCs, and Treg cells were obtained as detailed in Supplemental Figure 2. ConvT cells were stained with CTV and cultured in the bottom chamber un-stimulated or activated with Mitomycin-C treated-APCs plus soluble anti-CD3 antibody, in absence or presence of *wt, het*, or *Nrp1KO* Foxp3^YFP+^ Treg cells placed in the top chamber (*transwell*). ConvT cell proliferation was measured by tracking dye dilution by flow cytometry.

Supplemental Figure 4 Nrp1 expression on Treg and conventional T cells during allotransplant response. **(A)** Adoptive transfer and transplantation strategy. Responder CD45.1+ convT cells and CD45.2+ Treg cells from *wt, het*, and *Nrp1KO* animals were sort-purified as described in previous figures. RAG-KO recipient animals were i.v adoptively transferred with convT cells alone or with Treg cells. The next day, animals were transplanted with tail skin grafts from F1 animals (C57Bl/6 x Balb/c). Graft survival was monitored three times per week, and 20-days post-transplantation mice were euthanized and graft-draining lymph nodes (dLN) were harvested, stained with antibodies and analyzed by multi-parametric flow cytometry. **(B)** Total cell count from transplant-dLN. **(C)** Gating strategy for distinguishing between CD45.1+ cells (convT) and CD45.2+ cells (Treg cells). **(D)** Representative FMO negative control for Nrp1 (top) or Eos (bottom) on gated live CD4+ T cells from grafted mice dLN cells. **(E)** Representative contour plots depicting Nrp1 and Eos expression on gated live CD4+CD45.2+ Treg cells. **(F)** Accumulated frequency of Nrp1+Treg cells and **(G)** Eos+Treg cells from allografted RAG-KO mice receiving *wt, het*, or *Nrp1KO* Treg-treatment. Bars represent mean and each circle represents one mouse. For **(B,E,F)** Unpaired *T*-test, ^*^ < 0.05; ^**^ < 0.01; ^***^ < 0.001; ns, not significant. Data representative of at least two independent experiments with 4 mice per group.

## References

[1] Agarwal A Fanelli G Letizia M Tung SL Boardman D Lechler R. Regulatory T cell-derived exosomes: possible therapeutic and diagnostic tools in transplantation. Front Immunol. (2014) 5:555. 10.3389/fimmu.2014.00555 25414702 PMC4220709

[2] Josefowicz SZ Lu LF Rudensky AY. Regulatory T cells: mechanisms of differentiation and function. Annu Rev Immunol. (2012) 30:531–64. 10.1146/annurev.immunol.25.022106.141623 22224781 PMC6066374

[3] Romagnani S. Immunological tolerance and autoimmunity. Intern Emerg Med. (2006) 1:187–96. 10.1007/BF02934736 17120464

[4] Safinia N Scotta C Vaikunthanathan T Lechler RI Lombardi G. Regulatory T cells: serious contenders in the promise for immunological tolerance in transplantation. Front Immunol. (2015) 6:438. 10.3389/fimmu.2015.00438 26379673 PMC4553385

[5] Sanchez-Fueyo A Weber M Domenig C Strom TB Zheng XX. Tracking the immunoregulatory mechanisms active during allograft tolerance. J Immunol. (2002) 168:2274–81. 10.4049/jimmunol.168.5.2274 11859115

[6] Romano M Tung SL Smyth LA Lombardi G. Treg therapy in transplantation: a general overview. Transpl Int. (2017) 30:745–53. 10.1111/tri.12909 28012226

[7] Prud'homme GJ Glinka Y. Neuropilins are multifunctional coreceptors involved in tumor initiation, growth, metastasis and immunity. Oncotarget. (2012) 3:921–39. 10.18632/oncotarget.626 22948112 PMC3660061

[8] Lepelletier Y Smaniotto S Hadj-Slimane R Villa-Verde DM Nogueira AC Dardenne M. Control of human thymocyte migration by Neuropilin-1/Semaphorin-3A-mediated interactions. Proc Natl Acad Sci USA. (2007) 104:5545–50. 10.1073/pnas.0700705104 17369353 PMC1838472

[9] Tran DQ Shevach EM. Therapeutic potential of FOXP3(+) regulatory T cells and their interactions with dendritic cells. Hum Immunol. (2009) 70:294–9. 10.1016/j.humimm.2009.02.007 19236900 PMC11007672

[10] Oussa NA Dahmani A Gomis M Richaud M Andreev E Navab-Daneshmand AR. VEGF requires the receptor NRP-1 to inhibit lipopolysaccharide-dependent dendritic cell maturation. J Immunol. (2016) 197:3927–35. 10.4049/jimmunol.1601116 27815442

[11] Milpied P Massot B Renand A Diem S Herbelin A Leite-de-Moraes M. IL-17-producing invariant NKT cells in lymphoid organs are recent thymic emigrants identified by neuropilin-1 expression. Blood. (2011) 118:2993–3002. 10.1182/blood-2011-01-329268 21653940

[12] Tordjman R Lepelletier Y Lemarchandel V Cambot M Gaulard P Hermine O. A neuronal receptor, neuropilin-1, is essential for the initiation of the primary immune response. Nat Immunol. (2002) 3:477–82. 10.1038/ni789 11953749

[13] Sarris M Andersen KG Randow F Mayr L Betz AG. Neuropilin-1 expression on regulatory T cells enhances their interactions with dendritic cells during antigen recognition. Immunity. (2008) 28:402–13. 10.1016/j.immuni.2008.01.012 18328743 PMC2726439

[14] Delgoffe GM Woo SR Turnis ME Gravano DM Guy C Overacre AE. Stability and function of regulatory T cells is maintained by a neuropilin-1-semaphorin-4a axis. Nature. (2013) 501:252–6. 10.1038/nature12428 23913274 PMC3867145

[15] Hansen W Hutzler M Abel S Alter C Stockmann C Kliche S. Neuropilin 1 deficiency on CD4+Foxp3+ regulatory T cells impairs mouse melanoma growth. J Exp Med. (2012) 209:2001–16. 10.1084/jem.20111497 23045606 PMC3478934

[16] Jackson SR Berrien-Elliott M Yuan J Hsueh EC Teague RM. Neuropilin-1 expression is induced on tolerant self-reactive CD8+ T cells but is dispensable for the tolerant phenotype. PLoS ONE. (2014) 9:e110707. 10.1371/journal.pone.0110707 25343644 PMC4208794

[17] Gao YL Chai YF Qi AL Yao Y Liu YC Dong N Neuropilin-1highCD4(+)CD25(+) regulatory T cells exhibit primary negative immunoregulation in sepsis. Mediat Inflamm. (2016) 2016:7132158 10.1155/2016/7132158 PMC4863118 27239104

[18] Gao YL Yu MM Shou ST Yao Y Liu YC Wang LJ. Tuftsin prevents the negative immunoregulation of neuropilin-1highCD4+CD25+regulatory T cells and improves survival rate in septic mice. Oncotarget. (2016) 7:81791–805. 10.18632/oncotarget.13235 27835904 PMC5348430

[19] Fleissner D Hansen W Geffers R Buer J Westendorf AM. Local induction of immunosuppressive CD8+ T cells in the gut-associated lymphoid tissues. PLoS ONE. (2010) 5:e15373. 10.1371/journal.pone.0015373 20975955 PMC2958146

[20] Zhou H Zhang L Tong L Cai M Guo H Yang C. Expression of neuropilin-1 in kidney graft biopsies: what is the significance?. Transpl Proc. (2007) 39:81–3. 10.1016/j.transproceed.2006.10.221 17275479

[21] Yuan Q Hong S Shi B Kers J Li Z Pei X. CD4(+)CD25(-)Nrp1(+) T cells synergize with rapamycin to prevent murine cardiac allorejection in immunocompetent recipients. PLoS ONE. (2013) 8:e61151. 10.1371/journal.pone.0061151 23577203 PMC3618334

[22] Campos-Mora M Morales RA Pérez F Gajardo T Campos J Catalan D. Neuropilin-1 regulatory T cells promote skin allograft survival and modulate effector CD4 T cells phenotypic signature. Immunol Cell Biol. (2014) 93:113–9. 10.1038/icb.2014.77 25245111

[23] Kim HJ Barnitz RA Kreslavsky T Brown FD Moffett H Lemieux ME. Stable inhibitory activity of regulatory T cells requires the transcription factor Helios. Science. (2015) 350:334–9. 10.1126/science.aad0616 26472910 PMC4627635

[24] Rubtsov YP Rasmussen JP Chi EY Fontenot J Castelli L Ye X. Regulatory T cell-derived interleukin-10 limits inflammation at environmental interfaces. Immunity. (2008) 28:546–58. 10.1016/j.immuni.2008.02.017 18387831

[25] Sharma MD Huang L Choi JH Lee EJ Wilson JM Lemos H. An inherently bifunctional subset of Foxp3+ T helper cells is controlled by the transcription factor eos. Immunity. (2013) 38:998–1012. 10.1016/j.immuni.2013.01.013 23684987 PMC3681093

[26] Pan F Yu H Dang EV Barbi J Pan X Grosso JF. Eos mediates Foxp3-dependent gene silencing in CD4+ regulatory T cells. Science. (2009) 325:1142–6. 10.1126/science.1176077 19696312 PMC2859703

[27] Fan X Moltedo B Mendoza A Davydov AN Faire MB Mazutis L. CD49b defines functionally mature Treg cells that survey skin and vascular tissues. J Exp Med. (2018) 215:2796–814. 10.1084/jem.20181442 30355617 PMC6219731

[28] Rodríguez-Perea AL Arcia ED Rueda CM Velilla PA. Phenotypical characterization of regulatory T cells in humans and rodents. Clin Exp Immunol. (2016) 185:281–91. 10.1111/cei.12804 27124481 PMC4991523

[29] Spence A Purtha W Tam J Dong S Kim Y Ju CH. Revealing the specificity of regulatory T cells in murine autoimmune diabetes. Proc Natl Acad Sci USA. (2018) 115:5265–70. 10.1073/pnas.1715590115 29712852 PMC5960284

[30] Barthlott T Bosch AJ Berkemeier C Nogales-Cadenas R Jeker LT Keller MP. A subpopulation of CD103(pos) ICOS(pos) Treg cells occurs at high frequency in lymphopenic mice and represents a lymph node specific differentiation stage. Eur J Immunol. (2015) 45:1760–71. 10.1002/eji.201445235 25752506

[31] Annacker O Coombes JL Malmstrom V Uhlig HH Bourne T Johansson-Lindbom B. Essential role for CD103 in the T cell-mediated regulation of experimental colitis. J Exp Med. (2005) 202:1051–61. 10.1084/jem.20040662 16216886 PMC2213206

[32] Kobie JJ Shah PR Yang L Rebhahn JA Fowell DJ Mosmann TR. T regulatory and primed uncommitted CD4 T cells express CD73, which suppresses effector CD4 T cells by converting 5'-adenosine monophosphate to adenosine. J Immunol. (2006) 177:6780–6. 10.4049/jimmunol.177.10.6780 17082591

[33] Roberts V Stagg J Dwyer KM. The role of ectonucleotidases CD39 and CD73 and adenosine signaling in solid organ transplantation. Front Immunol. (2014) 5:64. 10.3389/fimmu.2014.00064 24600452 PMC3927137

[34] Komatsu N Okamoto K Sawa S Nakashima T Oh-hora M Kodama T. Pathogenic conversion of Foxp3+ T cells into TH17 cells in autoimmune arthritis. Nat Med. (2014) 20:62–8. 10.1038/nm.3432 24362934

[35] Yu F Sharma S Edwards J Feigenbaum L Zhu J Dynamic expression of T-bet and GATA3 by regulatory T cells maintains immune tolerance. Nat Immunol. (2015) 16:197–206. 10.1038/ni.3053 25501630 PMC4297509

[36] Lee GR. The balance of Th17 versus Treg cells in autoimmunity. Int J Mol Sci. (2018) 19:E730. 10.3390/ijms19030730 29510522 PMC5877591

[37] Amir AD Davis KL Tadmor MD Simonds EF Levine JH Bendall SC viSNE enables visualization of high dimensional single-cell data and reveals phenotypic heterogeneity of leukemia. Nat Biotechnol Res. (2013) 31:545–52. 10.1038/nbt.2594 PMC4076922 23685480

[38] Becher B Schlitzer A Chen J Mair F Sumatoh HR Teng KW. High-dimensional analysis of the murine myeloid cell system. Nat Immunol. (2014) 15:1181–9. 10.1038/ni.3006 25306126

[39] Huang YJ Haist V Baumgärtner W Föhse L Prinz I Suerbaum S. Induced and thymus-derived Foxp3(+) regulatory T cells share a common niche. Eur J Immunol. (2014) 44:460–8. 10.1002/eji.201343463 24170313

[40] Tatura R Zeschnigk M Hansen W Steinmann J Vidigal PG Hutzler M. Relevance of Foxp3(+) regulatory T cells for early and late phases of murine sepsis. Immunology. (2015) 146:144–56. 10.1111/imm.12490 26059660 PMC4552509

[41] Wang S Gao X Shen G Wang W Li J Zhao J. Interleukin-10 deficiency impairs regulatory T cell-derived neuropilin-1 functions and promotes Th1 and Th17 immunity. Sci Rep. (2016) 6:24249. 10.1038/srep24249 27075020 PMC4831052

[42] Battaglia A Buzzonetti A Monego G Peri L Ferrandina G Fanfani F. Neuropilin-1 expression identifies a subset of regulatory T cells in human lymph nodes that is modulated by preoperative chemoradiation therapy in cervical cancer. Immunology. (2008) 123:129–38. 10.1111/j.1365-2567.2007.02737.x 18028372 PMC2433274

[43] Piechnik A Dmoszynska A Omiotek M Mlak R Kowal M Stilgenbauer S. The VEGF receptor, neuropilin-1, represents a promising novel target for chronic lymphocytic leukemia patients. Int J Cancer. (2013) 133:1489–96. 10.1002/ijc.28135 23447383

[44] Overacre-Delgoffe AE Chikina M Dadey RE Yano H Brunazzi EA Shayan G. Interferon-gamma Drives Treg fragility to promote anti-tumor immunity. Cell. (2017) 169:1130–41.e11. 10.1016/j.cell.2017.05.005 28552348 PMC5509332

[45] Solomon BD Mueller C Chae WJ Alabanza LM Bynoe MS. Neuropilin-1 attenuates autoreactivity in experimental autoimmune encephalomyelitis. Proc Natl Acad Sci USA. (2011) 108:2040–5. 10.1073/pnas.1008721108 21245328 PMC3033275

[46] Smyth LA Ratnasothy K Tsang JY Boardman D Warley A Lechler R. CD73 expression on extracellular vesicles derived from CD4+ CD25+ Foxp3+ T cells contributes to their regulatory function. Eur J Immunol. (2013) 43:2430–40. 10.1002/eji.201242909 23749427

[47] Okoye S Coomes SM Pelly VS Czieso S Papayannopoulos V Tolmachova T MicroRNA-containing T-regulatory-cell-derived exosomes suppress pathogenic T helper 1 cells. Immunity. (2014) 41:89–103. 10.1016/j.immuni.2014.05.019 25035954 PMC4104030

[48] Aiello S Rocchetta F Longaretti L Faravelli S Todeschini M Cassis L. Extracellular vesicles derived from T regulatory cells suppress T cell proliferation and prolong allograft survival. Sci Rep. (2017) 7:11518. 10.1038/s41598-017-08617-3 28912528 PMC5599553

[49] Rieder SA Metidji A Glass DD Thornton AM Ikeda T Morgan BA. Eos is redundant for regulatory T cell function but plays an important role in IL-2 and Th17 production by CD4+ conventional T cells. J Immunol. (2015) 195:553–63. 10.4049/jimmunol.1500627 26062998 PMC4491037

[50] Gu C Rodriguez ER Reimert DV Shu T Fritzsch B Richards LJ. Neuropilin-1 conveys semaphorin and VEGF signaling during neural and cardiovascular development. Dev Cell. (2003) 5:45–57. 10.1016/S1534-5807(03)00169-2 12852851 PMC3918747

[51] Haribhai D Lin W Relland LM Truong N Williams CB Chatila TA. Regulatory T cells dynamically control the primary immune response to foreign antigen. J Immunol. (2007) 178:2961–72. 10.4049/jimmunol.178.5.2961 17312141

[52] Leelatian N Diggins KE Irish JM. Characterizing phenotypes and signaling networks of single human cells by mass cytometry. Methods Mol Biol. (2015) 1346:99–113. 10.1007/978-1-4939-2987-0_8 26542718 PMC4656023

[53] McMurchy AN Levings MK. Suppression assays with human T regulatory cells: a technical guide. Eur J Immunol. (2012) 42:27–34. 10.1002/eji.201141651 22161814

[54] Théry C Amigorena S Raposo G Clayton A Isolation and characterization of exosomes from cell culture supernatants and biological fluids. Curr Protoc Cell Biol. (2006) 30:3.22.1–29. 10.1002/0471143030.cb0322s30 18228490

[55] Lobb RJ Becker M Wen SW Wong CS Wiegmans AP Leimgruber A. Optimized exosome isolation protocol for cell culture supernatant and human plasma. J Extracell Vesicles. (2015) 4:27031. 10.3402/jev.v4.27031 26194179 PMC4507751

