# Supplementary figures and images for: CD4+Foxp3+T Regulatory Cells Promote Transplantation Tolerance by Modulating Effector CD4+ T Cells in a Neuropilin-1-Dependent Manner
Source: Front Immunol. 2019 Apr 24;10:882. doi: 10.3389/fimmu.2019.00882 (PMC6491519; doi:10.3389/fimmu.2019.00882)

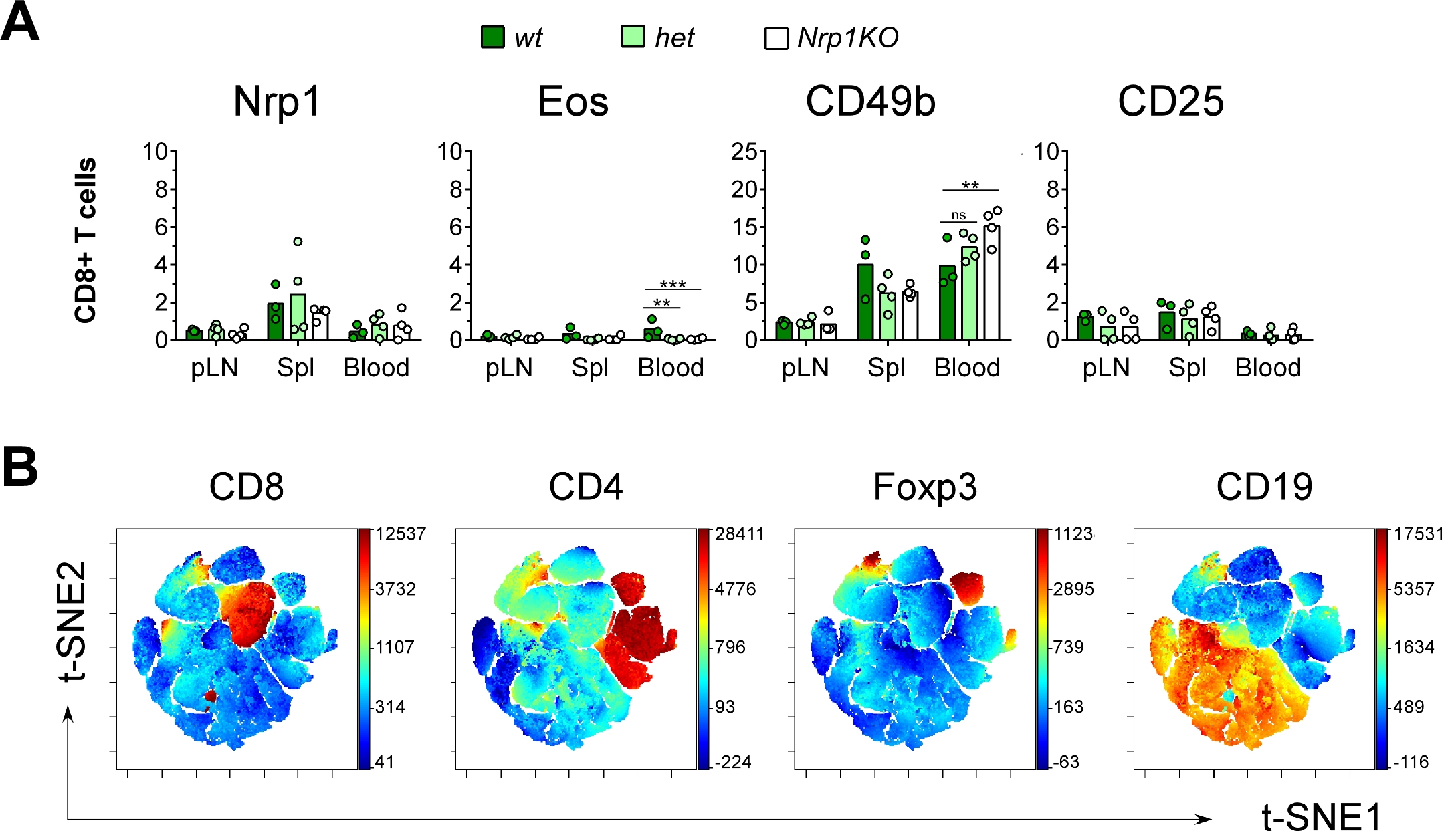

Supplement: Supplemental Figure 1 — viSNE defines lymphocyte subsets in lymphoid tissues from wt, het, and Nrp1KO mice. Organs and tissue were collected as described in Figure 1. (A) Leukocytes were obtained from wt (dark green bars), het (light green bars), and Nrp1KO (white bars) animals and tested for the indicated molecules expressed by CD8+ T cells. (B) viSNE heat maps show the expression of CD8, CD4, Foxp3, and CD19 in order to distinguish CD8+ T cells, CD4+Foxp3—convT cells, CD4+Foxp3+ Treg cells and CD4-CD8-Foxp3-CD19+ B cells. For (A), n = 3–4 animals per genotype, each circle represents one mouse, bars represent mean. Two-way ANOVA, ** < 0.01; *** < 0.001. For (B), X-axis corresponds to t-SNE1 parameter, left Y-axis corresponds to t-SNE2 parameter and right Y-axis indicates MFI for each marker for all the viSNE heat maps. Representative data of three independent experiments. [file Image_1.JPEG]

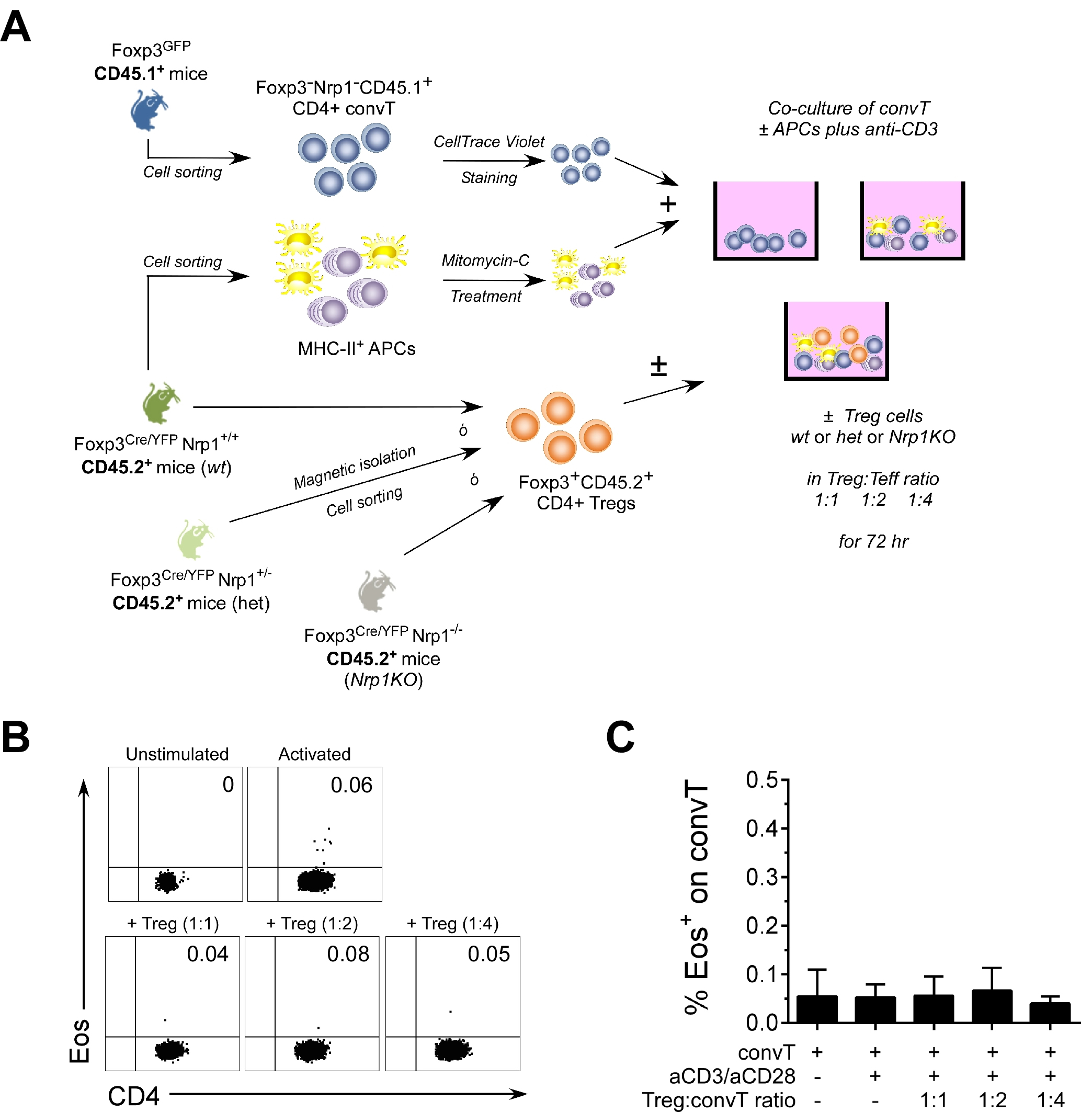

Supplement: Supplemental Figure 2 — Expression of Eos on conventional T cells upon contact-dependent culture with Treg cells. (A) Contact-dependent suppressive assay strategy: Responder convT cells (CD4+CD25-Foxp3GFP−Nrp1-CD45.1+) were sort-purified from Foxp3GFP+CD45.1+ animals, antigen presenting cells (or APCs, CD3-MHCII+CD45.2+), and Treg cells (CD4+Foxp3YFP+CD45.2+) were sort-purified from wt, het, and Nrp1KO-Foxp3YFP+animals. ConvT cells were stained with CellTrace™Violet and cultured un-stimulated or activated with Mitomycin-C treated-APCs plus soluble anti-CD3 antibody, in the absence or presence of wt, het, or Nrp1KO Foxp3YFP+ Treg cells. ConvT cell proliferation was measured by dye dilution using flow cytometry. (B) Representative dot plots show Eos expression on CD45.1+convT cells after 3 days of co-culture with wt Treg cells. (C) Accumulated frequency of Eos+ convT cells in the aforementioned conditions. For C, bars represent mean ± SEM, n = 2 independent experiments. [file Image_2.JPEG]

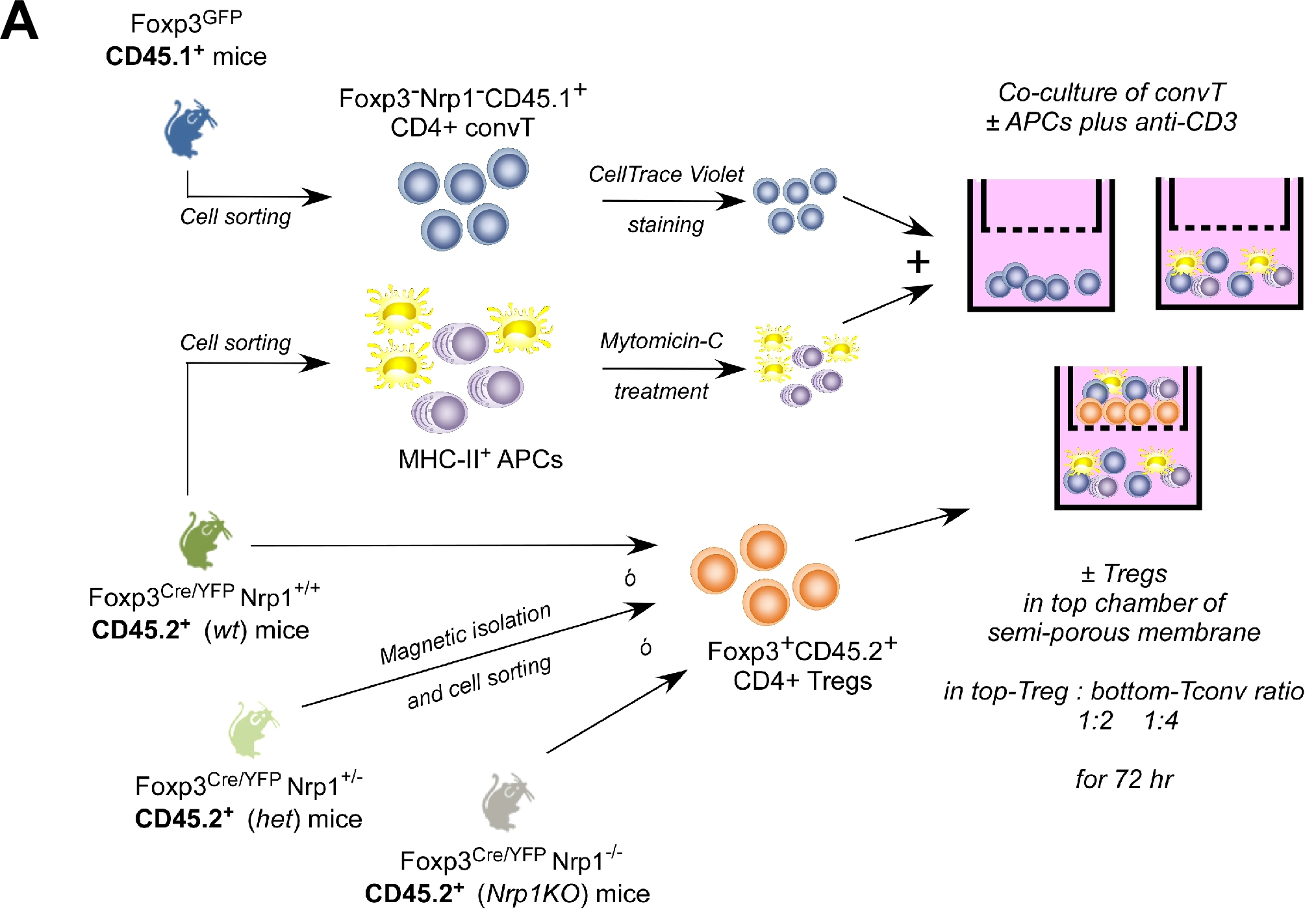

Supplement: Supplemental Figure 3 — Contact-independent Treg cell suppression assay. Contact-independent suppressive assay strategy: responder convT, APCs, and Treg cells were obtained as detailed in Supplemental Figure 2. ConvT cells were stained with CTV and cultured in the bottom chamber un-stimulated or activated with Mitomycin-C treated-APCs plus soluble anti-CD3 antibody, in absence or presence of wt, het, or Nrp1KO Foxp3YFP+ Treg cells placed in the top chamber (transwell). ConvT cell proliferation was measured by tracking dye dilution by flow cytometry. [file Image_3.JPEG]

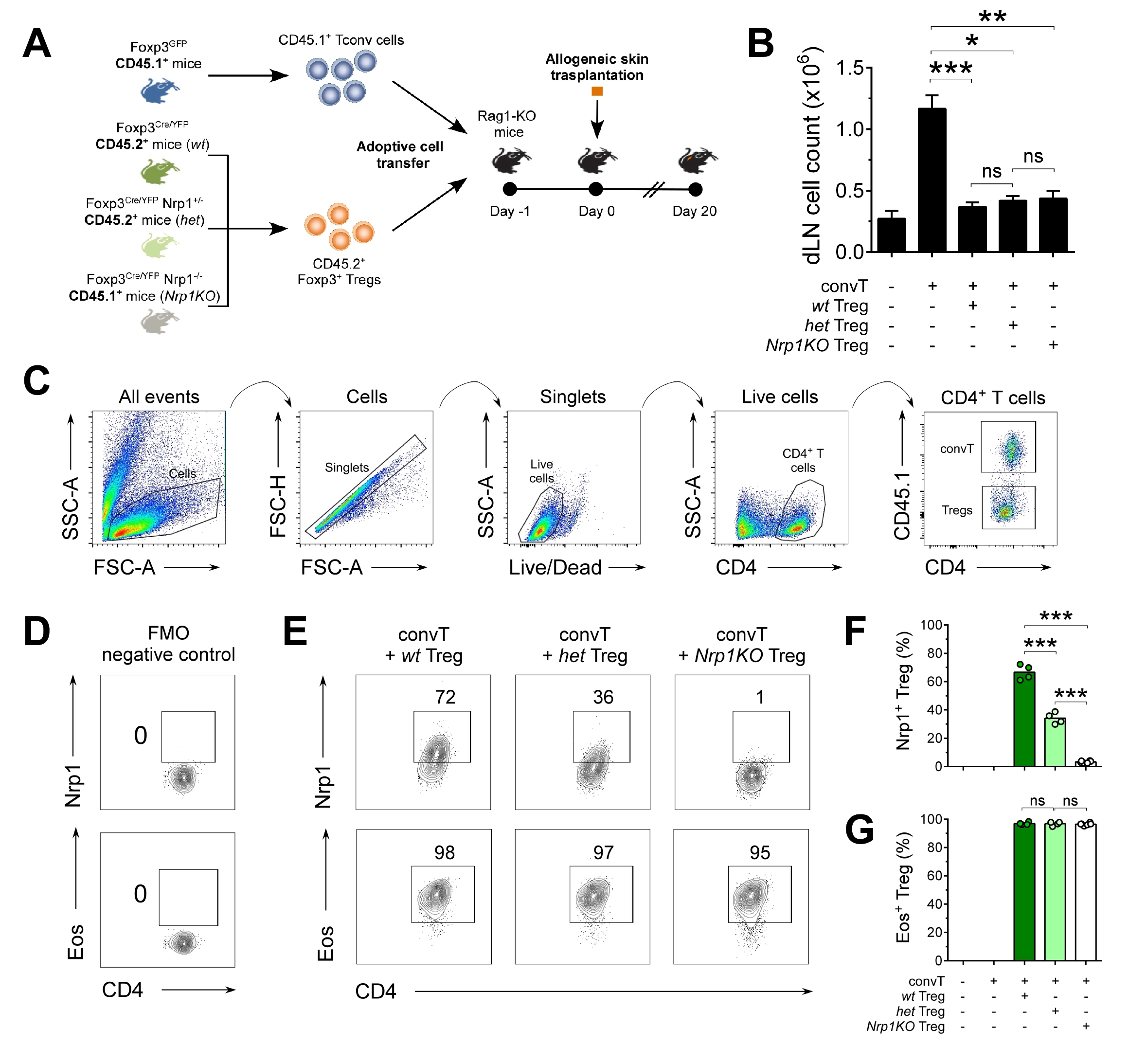

Supplement: Supplemental Figure 4 — Nrp1 expression on Treg and conventional T cells during allotransplant response. (A) Adoptive transfer and transplantation strategy. Responder CD45.1+ convT cells and CD45.2+ Treg cells from wt, het, and Nrp1KO animals were sort-purified as described in previous figures. RAG-KO recipient animals were i.v adoptively transferred with convT cells alone or with Treg cells. The next day, animals were transplanted with tail skin grafts from F1 animals (C57Bl/6 x Balb/c). Graft survival was monitored three times per week, and 20-days post-transplantation mice were euthanized and graft-draining lymph nodes (dLN) were harvested, stained with antibodies and analyzed by multi-parametric flow cytometry. (B) Total cell count from transplant-dLN. (C) Gating strategy for distinguishing between CD45.1+ cells (convT) and CD45.2+ cells (Treg cells). (D) Representative FMO negative control for Nrp1 (top) or Eos (bottom) on gated live CD4+ T cells from grafted mice dLN cells. (E) Representative contour plots depicting Nrp1 and Eos expression on gated live CD4+CD45.2+ Treg cells. (F) Accumulated frequency of Nrp1+Treg cells and (G) Eos+Treg cells from allografted RAG-KO mice receiving wt, het, or Nrp1KO Treg-treatment. Bars represent mean and each circle represents one mouse. For (B,E,F) Unpaired T-test, * < 0.05; ** < 0.01; *** < 0.001; ns, not significant. Data representative of at least two independent experiments with 4 mice per group. [file Image_4.JPEG]
